# Factors influencing dropout rate of intermittent preventive treatment of malaria during pregnancy

**DOI:** 10.1186/s13104-016-2265-2

**Published:** 2016-10-10

**Authors:** David Teye Doku, Mumuni Mukaila Zankawah, Addae Boateng Adu-Gyamfi

**Affiliations:** 1 Department of Population and Health, Private Mail Bag, University of Cape Coast, Cape Coast, Ghana; 2 School of Health Sciences, University of Tampere, Tampere, Finland; 3 Metropolitan Health Directorate, Tamale, Ghana

**Keywords:** Intermittent preventive treatment, Dropout rate, Malaria in pregnancy

## Abstract

**Background:**

The burden of malaria in terms of morbidity and mortality is huge is Sub-Saharan Africa, particularly among pregnant women. Among the measures to curb down this burden include intermittent preventive treatment (IPT) and effective case management. These strategies were adopted by Ghana and implemented since 2003; however, there is still high dropout rate in IPT coverage. This study sought to investigate factors contributing to high dropout rate between IPT1 and IPT3 in the Tamale Metropolis, one of the health facilities with the highest IPT dropout rates in Ghana.

**Methods:**

Survey, in-depth interviews and short ethnographic techniques were conducted among pregnant women, antenatal care (ANC) health workers and heads of health facilities to investigate factors which account for dropout rate of intermittent treatment of malaria.

**Results:**

Shortage of sulphadoxine pyrimethamine (SP), inadequate supply of portable water for administration of SP, unavailability of IPT during outreach services, lack of knowledge by ANC staff about the dropout rate in their area of jurisdiction and poor attitude of some health workers were identified as barriers to achieving high IPT3 coverage.

**Conclusions:**

Late ANC visit, provider and logistical barriers account for the women’s missed opportunities to prevent malaria in pregnancy through IPT. Addressing the above barriers will contribute to saving lives and ensuring progress towards the goal of combating malaria as well as reducing maternal, neonatal and child mortalities.

**Electronic supplementary material:**

The online version of this article (doi:10.1186/s13104-016-2265-2) contains supplementary material, which is available to authorized users.

## Background

The toll of malaria is estimated at 300 million cases each year worldwide of which more than 90 % occur in Sub-Saharan Africa [1]. Approximately fifty million women living in malaria endemic countries throughout the world become pregnant, of whom over half live in tropical areas of Africa with intense transmission of *plasmodium falciparum* [2]. An estimated ten thousand of these women and two hundred thousand of their infants die as a result of malaria infection during pregnancy [2].

Malaria is a complex public health problem in African, where most cases and deaths due to the disease occur. An estimated 74 % of the population in the African region live in areas that are highly endemic for malaria [3]. Maternal malaria is associated with serious adverse pregnancy outcomes such as maternal deaths, maternal anaemia and low birth weight in most malaria endemic countries. Among pregnant women, malaria accounts for 13.8 % of out patient department attendance, 10.6 % of admissions and 9.4 % of deaths [4]. The African Summit on Roll Back Malaria in April 2000 adopted the Abuja Declaration in which regional leaders committed themselves to ensuring that 60 % of pregnant women in malaria-endemic communities accessed effective prevention and treatment of malaria by 2005 [5].

One major recommended means of preventing malaria during pregnancy is intermittent preventive treatment (IPT) with sulphadoxine pyrimethamine (SP) as a prophylactic treatment strategy to help reduce the severe burden and disastrous effects of malaria on pregnant women and their unborn babies [1]. The World Health Organisation (WHO) recommended that the policy for the prevention of malaria during pregnancy in areas of stable transmission should emphasize a preventive package of intermittent preventive treatment (IPT) and Insecticide Treated bed Nets and ensure effective case management of malaria illness [6].

The IPT package ensures that all pregnant women in areas of stable malaria transmission receive at least two doses of IPT after quickening, that is, after 16 weeks of gestation. Secondly, a schedule of four ANC visits should be available for a pregnant woman with three occurring after quickening with the delivery of IPT at each scheduled visit to ensure that a high proportion of women received at least two doses of IPT.

Intermittent Preventive Treatment of malaria in pregnancy using SP approach has been shown to be safe, inexpensive and effective. A study by Verhoeff et al. [7] in Malawi evaluating IPT showed a decline in placental malaria infection (32–23 %) and in the number of low birth weight babies (23–10 %). In Ghana, the recommended doses for IPT is three doses of SP as directly observed treatment (DOT) following screening for allergy and other reactions to sulfa drugs [1]. IPT with SP is provided as part of a comprehensive antenatal package to control maternal anaemia. Intermittent preventive treatment with SP for malaria in pregnancy in areas of high or seasonal malaria transmission has been shown to increase both maternal haemoglobin levels and the infant’s birth weight [8–11]. Similarly, Katsurangawa et al. [12] found that IPT reduced relapses and recurrence of malaria in children in Brazil.

Ghana adopted IPT with SP as a policy in 2003 in 20 selected districts but in 2005, it was scaled up nationwide to all districts to help reduce the severe burden and damaging effects of malaria morbidity and mortality on the pregnant woman and the unborn baby [1, 5].

The drug of choice for IPT in Ghana is SP because of its safety, effectiveness in reproductive-age women, and the feasibility for use in programmes as it can be delivered as a single-dose treatment under direct observation (DOT) by the health worker [1]. In addition to the above, SP is inexpensive, and has demonstrated high levels of acceptance by pregnant women [4]. Its administration twice in pregnancy has been found to reduce maternal anaemia by 39 % [13], placental malaria by 56 %, and low birth weight by 43 % [14].

Statistics available at the Tamale Metropolitan Health Directorate in Ghana prior to this study indicates that there is a high dropout rate in IPT coverage, especially between IPT1 and IPT3 in the Metropolis. The dropout rates between IPT1 and IPT3 recorded by year were: 2006–56.2 %, 2007–46.9 %, 2008–46 % and 2009–43.7 % while between IPT1 and IPT2 the following were recorded; 2006–33.3 %, 2007–25.5 %, 2008–23.3 % and 2009–37.8 [15]. Although there has been a general improvement of IPT coverage over the years, the dropouts remained generally high, especially between IPT1 and IPT3.

Due to the numerous advantages associated with the use of IPT during pregnancy, one would have thought that all pregnant women would adhere to its use. However, there is a high dropout rate in Ghana because of delay in commencement of SP which consequently reduces the number of doses a woman receives IPT during pregnancy. The age of pregnancy at first ante natal clinic (ANC) attendance is thus very crucial if a pregnant woman is to obtain all the recommended three doses of SP. van Eijk et al. [10] observed that late first ANC attendance and the level of formal education of pregnant women contribute to incomplete IPT. Anders et al. [16] have argued that some of the causes of the late attendance include women who encounter no problems during pregnancy and therefore see no need to visit the clinic. Other factors reported in previous studies include travel distance from home to the clinic and inability to leave farm work to travel to the clinics [16, 17]. Mubyazi et al. [17], argue that a number of pregnant women attend ANC before 16 weeks gestation when they are not due to be given SP at that first visit, but do not return for their first dose SP at the recommended time. For example, in a study by Anders et al. [16], almost half (48 %) of all respondents had attended ANC at or before 4 months of gestation, however 86 % of these early attendees did not receive IPT on their first visit.

Some studies have found that the uptake of preventive treatment of malaria in pregnancy using SP increases with higher levels of formal education [18–20]. On the contrary, a study done in Tanzania by Marchant et al. [21] showed no evidence of an association between any individual factors and second dose coverage beyond living in an urban area. Age, marital status, educational level of the woman and household socioeconomic status were all not associated with second dose coverage of SP. However, Acquah [22] illustrated that there is an association between IPT uptake and marital status, employment, maternal educational level, use of ANC services, gestational age at first ANC visit and maternal knowledge about malaria was statistically significant. The goal of this study was to identify factors which contribute to the high dropout rate between IPT1 and IPT3 in the Tamale Metropolis in Ghana.

## Methods

### Study design

Survey, in-depth interviews and short ethnographic technique were the main methods employed in this study.

### Study area

The study was conducted among 294 respondents in the Tamale Metropolis in 2011 over a period of 6 weeks.

### Study population

The sampled population included pregnant women whose gestation exceeded 36 weeks at antenatal clinics (ANC), postnatal mothers whose children were less than 12 weeks old at Child Welfare Clinic (CWC) and health workers comprising heads of health facilities, midwives, general nurses, medical assistants and community health nurses. The inclusion of only pregnant women whose gestation exceeded 36 weeks was necessary in this study because they were all expected to have completed the 3rd dose of IPT and SP. One hundred and four (104) general nurses, mid-wives, nursing assistants, medical assistants, community health nurses and public health nurses and six heads of facilities were also included in the study as they were the health staff that administered the SP and would therefore be able to identify the administrative bottlenecks that may cause the dropout between IPT1 and IPT3.

### Sampling and sample size

Respondents were drawn from all the six Sub Districts in the Tamale Metropolis in proportion to their population sizes. Simple random sampling was used to select 184 pregnant women, postpartum mothers and health staff. In addition, a total of 104 ANC workers comprising of general nurses, mid-wives, nursing assistants, medical assistants, community health nurses and public health nurses were randomly selected from six health facilities in the six districts within the Tamale Metropolis. The six heads of the selected health facilities were also purposively selected and interviewed.

### Data collection

Structured questionnaire and observation guide were used for the data collection. The first section of the structured questionnaire had questions on the description of the Sub District where the data were collected, including the description of the health facility. The second part had questions on the socio-demographic background of the respondents, the third part focused on the effects of malaria on the pregnant woman and the foetus, the awareness, knowledge and perception of respondents on intermittent preventive treatment of malaria in pregnancy, opinion of the respondents on how to motivate mothers to visit the ANC regularly to take SP as part of the IPT schedule, while the fifth section was on the attitude of the health staff towards the pregnant women. The instrument was pre-tested among a sample of the target population in one of the health facilities in the region but which was not part of the sampled health facilities. Structured questionnaires were administered to the heads of the health facilities, medical assistants, nurses and midwives as well as nursing mothers at the CWCs and pregnant women at ANCs. The ANC cards of all pregnant women and postpartum mothers were inspected and qualified respondents (pregnant women whose gestation exceeded 36 weeks and mothers who are less than 12 weeks after delivery) were identified.

Besides, non-participant observation was employed at the ANC’s to investigate the practices and availability of logistics for the IPT implementation as well as the attitude of health workers toward daily administration of the IPT. Non participant observation guide included, name of facility, sub-district, health education programme drawn for the quarter including malaria in pregnancy, health education programme drawn for the quarter include intermittent preventive treatment of malaria in pregnancy, whether there were visible signs of posters of IPT posted on the wall, availability of SP available at ANC (health workers issuing SP to clients), clients swallowing SP before health workers, ANC staff recording SP given in ANC book of clients, the availability of free safe and clean water for DOT at ANC. The instrument for the data collection is appended to this report as Additional file 1. The data collection was conducted with assistance of 12 students from the Tamale School of Hygiene who were fluent in Dagbani (the main language spoken in the area). These assistants were mainly engaged in the administration of the questionnaire. The rationale of the study was explained to the respondents and they assured of confidentiality of their responses as well as the option to opt out at stage of the questionnaire administration or refuse to response to any question(s) which they do not wish to answer. Observations were recorded in the investigator’s notebook. The qualitative data presented in the report were mainly from the observations. The institutional review board, through the Population and Health Departmental Board of the Faculty of Social Sciences of the University of Cape Coast, Ghana, gave the ethical clearance for the study. Verbal consents were obtained from participants and for minors their parents, guidance, or other surrogate adults gave consent on their behalf.

### Data analysis

In-depth interviews and observations were analysed using ethnographic technique by identifying categories and concepts from the data as they emerged. Content analysis involving systematic scrutiny of field notebooks and derivation of understanding was also used to analyse the data. The survey part of the data was presented as frequency in relation to the qualitative findings.

## Results

The socio-demographic characteristics of respondents are presented in Table 1. Women aged 25–29 were the largest age group. Nearly all the women were married (95.1 %). Those without any formal education and Moslems were the major educational and religious categories, respectively. Furthermore, traders and those with two or more children were the major occupational and parity categories, respectively (Table 1).

**Table 1:** The socio-demographic characteristics of respondents

Age	Frequency	Percentage
15–19	7	3.7
20–24	41	22.3
25–29	62	33.7
30–34	38	20.7
35–39	25	13.6
40+	11	6
Marital status		
Married	175	95.1
Single	5	2.7
Co-habitation	4	2.2
Level of education		
No forma education	82	44.5
Primary	34	18.5
JHS/JHS/Middle School	20	10.9
SSS/SHS	37	20.1
Technical/vocational	2	1.1
Tertiary	9	4.9
Religion		
Islam	144	78.3
Christianity	34	18.5
Traditional	3	1.6
Others	3	1.6
Occupation		
Government workers	19	10.3
Farmers	33	17.9
Traders	61	33.3
Food processors	25	13.6
Seamstress	21	11.4
Hair dressers	17	9.2
Others	8	4.3
Parity		
Nil	19	12.4
1–2	77	41.8
Above 2	88	45.8

We investigated the administration of SP at the health facilities and found that some of the pregnant women were not given SP at their first visit. Thirty-two percent of pregnant women (58/184) indicated that they were not given SP during their first ANC visit while 25.5 % (46/184) did not take SP during the subsequent visit.

The respondents sampled for the study were interviewed on the number of doses of SP taken and their responses are represented in Table 2. Eighty-five percent of the respondents received at least one dose of SP, 8.7 % of the respondents have received one dose of SP, and 35.3 % of them received two doses while 38 % received three doses.

**Table 2:** Number of SP doses taken

No. of doses of SP taken	Frequency	Percentage
Once	16	8.7
Twice	65	35.3
Thrice	70	38.0
Can’t remember	33	17.9
Total	184	100

It was observed that most health facilities did not have clean water supply. Sixty-seven percent of the respondents confirmed that there was no water at the health facilities for the practice of DOT. About 5.4 % of the respondents had their own water, 7.6 % of the respondents said they fetched the water from a tap within the health facility, 40.7 % of them bought the water and 0.5 % had water from other sources.

It was observed that, generally, the attitude of health workers was acceptable. Respondents also indicated that reception by staff of the health facilities was good and the services they rendered were satisfactory. Despite this, some women (32/184) indicated that they were shouted at the health facility whiles nearly one out of ten said they would not ask health workers questions because they (health workers) did not behave well towards them (pregnant women) Tables 3 and 4.

**Table 3:** Attitude of staff

Attitude	Frequency	Percentage
Good reception by staff	177	96.2
Not asked questions due to bad behaviour	16	8.7
Shouted at	32	17.4
Questions satisfactorily answered	127	69
Satisfactory service	167	90.8
Good behaviour by staff	120	65.2

**Table 4:** Knowledge of ANC staff on IPT

Component of knowledge	Frequency	Percentage
Correct def. of IPT	94	85.5
Recommended drug for IPT	97	88.2
When not to give IPT	64	58.2
Recommended doses for IPT	99	90
Correct interval for IPT	103	93.6
Side effects of SP	79	71.8

We inquired from the women suggestions to improve the coverage of IPT2 and IPT3, and 18.5 % of the them said the provision of sachet water was needed, 17.9 % indicated respect for clients, 27.7 % said further education on IPT was needed, 14.1 % said motivation of mothers was needed to take IPT instead of shouting at them while 21.7 % gave other reasons.

When ANC staff’s knowledge regarding the use of IPT was investigated, we found that 85.5 % of ANC of them could give the right definition of IPT, 88.2 % knew of the recommended drug for IPT in Ghana, 58.2 % knew when to stop administering IPT, 90 % of the ANC staff knew the recommended doses for IPT, 93.6 % knew the correct interval for administering IPT and 71.8 % knew the side effects.

Ninety percent of the ANC staff reported that they observed clients swallowing the SP at the facility. However, about 56 % of the staff sampled indicated they had run short of SP at their facilities. Furthermore, half (50.9 %) of the health facilities do not provide IPT services during outreach. There was poor monitoring as a third (33.6 %) of the facilities were not monitored. Aside the poor monitoring, on the coverage, only a few (5.5 %) of the ANC staff were aware of the expected or acceptable margin of dropout rate.

An observation guide was used to determine the level of health education on malaria in general, malaria in pregnancy, intermittent preventive of malaria in pregnancy on the health education roster of various health facilities as well as whether the health workers who administered the SP educated the pregnant women on the IPT schedules. The findings show that most of the facilities visited had malaria on their quarterly health education schedule. However, none of the facilities factored in malaria in pregnancy and intermittent preventive treatment of malaria in pregnancy in their quarterly health education programme.

## Discussion

A number of factors have been observed or raised by respondents as influencing the administration of IPT at the health facilities. These factors can either positively or negatively affect the IPT administration process. Some of these factors include the poor administration of SP at the facility, improper planning of stock management leading to the shortage of SP, health workers’ knowledge on IPT, health workers’ attitude towards client, unavailability of IPT services during outreach activities and health workers themselves giving wrong messages about the timing of the administration of the drug could undermine the achievement of IPT3 coverage.

A third of respondents stated that they had not taken SP during their first visit while a quarter also reported they did not take it in the subsequent visit. This might be so because some of the respondents visited the ANC before 16 weeks and therefore were not due for SP, it could also be associated with the shortages of SP as indicated by the ANC staff. If it is due to shortage, pregnant women may become reluctant in visiting ANC.

The recommended doses of IPT for pregnant women in Ghana are three doses in order to realise the full benefits of the IPT. Nevertheless, less than half of pregnant women reported receiving IPT three times during pregnancy. Our finding of less than three doses of IPT by some pregnant women is similar to those found in Uganda [23] where it was reported that only 35.8 % of women received two or more doses of SP.

Directly observed treatment (DOT) requires that SP be administered under the direct observation of a health worker. This is a sure way of ensuring that the pregnant woman takes the SP. The availability of water is one of the major necessary logistics needed to practice DOT for IPT. Taking SP requires the availability of water at the facility especially to ensure that the client take SP at the facility under observation as required by the IPT programme in Ghana instead of taking it at home. In this study, we observed that water was not available at most of the health facilities for pregnant women to take the SP. A number of the women bought their own water at the health facility to take SP. This could discourage some women from visiting ANC especially those who may not have money to buy the water even though all other antenatal services were offered free of charge. The unavailability of water at most of the facilities could also lead to low intake of SP and consequently high dropout rate. In Ghana, a study by President’s Malaria Initiative (PMI) [24] cited health workers as contributing to missing the opportunity to get all pregnant women who visit hospital to get IPT. Poor health worker practices have also been identified among others as operational challenges in delivering IPT [25].

In the Korogwe district of north-east Tanzania, Mubyazi et al. [17] found that only a quarter of pregnant women took SP under observation by a health worker. In this study, nine out of every ten ANC health worker reported that they observed the women to swallow the medicine thus higher than what was reported in Tanzania.

When clients’ views were sought on what should be done to improve the coverage of IPT2 and IPT3, the provision of sachet water, respect for clients, further education on IPT and motivation of mothers to take IPT instead of shouting at them were the main suggestions which emerged. The views expressed by the respondents are essential and should be factored into interventions which seek to address the high IPT dropout rates. It was observed that, although, the attitude of staff towards the pregnant women was generally acceptable, the attitude of some needs to be improved in order not to discourage the women from patronising ANC services.

The successful implementation of the IPT strategy relies on the level and type of training given to the health staff. A study by Brabin et al. [26] in the Gambia showed that women relied on health workers to provide safe drugs at the correct time. Without good training and close supervision of health care workers, there is the risk that pregnant women may not be treated well or health workers may shirk their responsibilities. The ANC staff knew the recommended doses for IPT, and nine out of ten knew the correct interval for administering IPT as well as the side effects. This is an indication that the knowledge of health workers in Tamale Metropolis is high, however, this does not reflect on the knowledge level of the women in describing the actual concept of IPT and the use of IPT in preventing malaria. This confirms the lack of health education for the pregnant women, which was also observed in this study.

About half of the health facilities did not provide IPT services during outreach activities in the communities. The shortage of SP, suspension of IPT programme during shortage and non-provision of IPT services during outreach could contribute to the high dropout rate between IPT1 and IPT3. According to ANC staff, IPT services virtually came to a halt anytime there was a shortage. A study by Tarimo [27] reported that 40 % of women interviewed in Tanzania had not received SP because of SP unavailability, suggesting that stock shortage is a major barrier to malaria prevention in the region.

Monitoring and supervision ensures that logistics and human resources needed for the implementation of the IPT programme at the facility level are made available at the appropriate times and used judiciously for their intended purposes. It also provides the opportunity for identifying deficiencies and urgently addressing them to ensure a continuity of the programme and achievement of its objectives. We found that monitoring of the IPT was poor, implying that most of the ANC staff were unable to track their IPT coverage and hence will be less likely to identify the high dropout rates early and intervene. It is possible that the inadequate monitoring of the IPT activities has also contributed to the high dropout rate in the Tamale Metropolis.

## Conclusion

Late ANC visit, shortage of SP, provider attitude and poor knowledge of pregnant women on IPT for malaria treatment might have contributed to the high IPT dropout rates. Logistics for IPT are necessary for its continuity. There should be a planned schedule for the procurement of SP to avoid shortages that usually lead to the suspension of the whole IPT programme in some of the health facilities. Efforts should also be made to provide portable water at ANC to be used by the clients to take SP under DOT, since DOT is necessary in order to measure the coverage of  the IPT. As IPT is a free service, the government of Ghana and other benevolent organisations should give financial assistance to health service providers to ensure the availability of SP and continuity of the IPT services. Such aid will for example help providers procure the necessary logistics to avoid the shortages of SP. It will also help in the provision of portable water to ensure the practice of DOT.

Special education should be given to ANC staff on the key monitoring indicators such as measurement of coverage and dropout rates. A system should be developed to aid IPT providers to track and monitor IPT coverage in their areas of operation, preferably monthly. This will help ANC staff to monitor their performance, identify problems and take immediate actions to address them.

## Additional file

10.1186/s13104-016-2265-2 Questionnaire, interview and observational guides for data collection on intermittent preventive treatment for malaria dropout rate.

## Abbreviations

ANC: antenatal care
CWC: Child Welfare Clinic
DOT: direct observed therapy
IPT: intermittent preventive treatment
PMI: President’s Malaria Initiative
SP: sulphadoxine pyrimethamine
WHO: World Health Organization
