# Supplementary material for: Factors influencing dropout rate of intermittent preventive treatment of malaria during pregnancy
Source: BMC Res Notes. 2016 Oct 10;9:460. doi: 10.1186/s13104-016-2265-2 (PMC5057376; doi:10.1186/s13104-016-2265-2)
Supplement: Supplementary file 1 — 10.1186/s13104-016-2265-2 Questionnaire, interview and observational guides for data collection on intermittent preventive treatment for malaria dropout rate. [file 13104_2016_2265_MOESM1_ESM.doc]

**University of Cape Coast**

**Faculty of Social Sciences**

**Department of Population and Health**

**Questionnaire for Pregnant women and Postpartum mothers**

Questionnaire Code No:

1. Sub-district: ………………………
2. Health facility………………………………………………………………

**SECTION1: SOCIO-DEMOGRAPHIC FACTORS**.

1. Age: ...........................
2. Place of residence: …………………………
3. Number of week’s gestation: .........................
4. Age of child........................
5. Gravidity (number of pregnancies).....................:
6. Parity (number of births): .................
7. No of live births: ................................
8. Marital Status: A. Married { } B. Single { } C. Widowed { } D. Co-habiting { } E. Divorced { }
9. Religion A. Islam { } B. Christianity { } C. Traditional religion D. Other (s) specify.......................
10. Level of education: A. No education { } B. Primary { } C. JHS/Middle school { } D. SHS { } D. Technical/Vocational { } D Tertiary { } E. { } F. Other(s), specify……….........
11. Occupation: A. Government worker { } Farmer { } B. Trader {} C. Food processor { } D. Seamstress {} E. Hair dresser { } F. Other(s), specify: …………
12. How long does it take you to get to the clinic? A. Less than 30 minutes B. Between 30 minutes to 1 hour { } C. More than 1 hour { }
13. How far is the place from a hospital or clinic from? A. Less than 1km {} B. Between 1-2km C. More than 3km {}
14. By what means of transport do you get to the health facility A. Walking { } B. Moto-bike { } C. Taxi { } D. Personal car { } E. Other(s) specify...................
15. Can the distance, time, and means of getting to the health facility prevent you from visiting the health facility regularly to access services? A. Yes { } B. No { }

**SECTION 2: Awareness and knowledge level of respondents on Intermittent Preventive Treatment of Malaria in Pregnancy**

1. Are you aware you can prevent yourself and your unborn child from malaria during pregnancy using some drugs? A. Yes { } B. No { }

**If your answer to question 11 is no skip to question 14**

1. If your answer to question 11 is ‘yes’, where can you acquire the drugs? A. Health facility { } B. Chemical shop { } C. Drug peddlers { } E. Traditional Birth Attendant (TBA) { } F. Village Volunteer { } G. Herbalist { } H. Other(s) specify........................
2. If your answer to question 11 is ‘yes’, what is the source of your information about where you can acquire the drugs?

A. ANC staff at ANC { } B. Radio { } C. Television { } D. Mobile health service van { } E. Other pregnant women { } F. other, specify ……………

1. Have you heard of Intermittent Preventive Treatment (IPT)? A. Yes { } B. No { }
2. If your answer to question 13 is ‘yes’, what is Intermittent Preventive Treatment (IPT)?
3. A. The use of some medicines to prevent malaria during pregnancy

B. Preventing illnesses during pregnancy.

C. Eating good foods to remain healthy.

D. Visiting the health facility for regular check ups

1. Have you heard of the Sulphadoxine Pyrimethanie (SP) drug? (‘Tim kpila dibata’) A. Yes { } B. No { }

**If your answer to question 22 is ‘no’, then skip to question 31**

1. If your answer to question 22 is ‘yes’ do you know what the tablets (Sulphadoxine Pyrimethanie) are used for? A. Yes { } B. No { }

**If your answer to question 23 is ‘no’ then skip to question 25**

1. If your answer to question 23 is ‘yes’, what is the tablet used for?

A. To make me gain weight { }

B. To make my baby and I strong and healthy { }

C. To prevent me from getting malaria { }

D. To give me a lot of blood { }

E. Other(s) specify …………………………

1. How many times should a pregnant woman swallow the tablets Sulphadoxine Pyrimethamine (SP) at Ante-natal clinic (ANC) during pregnancy? A. Once { } B. Twice { } C. Thrice { } D. Don’t know { }
2. What is the minimum number of times during pregnancy that a pregnant woman should swallow the tablets? A. Once { } B. Twice { }
3. At what age of gestation should a pregnant woman start taking Sulphadoxine Pyrimethamine (SP)? A. Before quickening starts { } B. At 16 weeks { } C. One month after conception { } D. After quickening { } E. After 36 weeks { }
4. Is there a pattern for taking Sulphadoxine Pyrimethamine (SP) Drugs? A. Yes { } B. No { }

**If your answer to question 28 is ‘no’ them skip to question 32**

1. If your answer to question 28 is ‘yes’, what is the pattern, schedule or interval that the pregnant woman should take the medicine at the Ante-natal clinic? A. Weekly { } B. Every two weeks { } C. Monthly { } D. Don’t know { }
2. Do you think that Intermittent Preventive Treatment has some benefit? A. Yes { } B. { }

**If your answer to question 30 is ‘no’, then skip to question 32**

1. If your answer to question 30 is ‘yes’, mention some of the benefits of Intermittent Preventive Treatment.

A. Prevent anaemia in pregnancy

B. Reduces maternal deaths { }

C. Reduces infant deaths { }

D. Prevent maternal malaria { }

E. Improves maternal weight { }

**Section 3: Knowledge of respondents on the effects of malaria in pregnancy**

1. Have you heard of malaria? A. Yes { } B. No { }

**If your answer to question 32 is ‘no’ skip to question 35**

1. If your answer to question 32 is ‘yes’, do you think malaria has some effects on the pregnant woman and the unborn child? A. Yes { } B. No { }

**If your answer to question 32 is ‘no’ skip to question 32**

1. If your answer to question 28 is ‘yes’, what are some of the effects of malaria on the pregnant woman? A. Anaemia { } B. Death {} C. Weaken her { } D. Nothing { } E. Don’t know { } F. Other, specify...
2. What are the effects of malaria on the unborn baby? A. Spontaneous abortion { } B. Intra Uterine Death { } C. Low birth weight { } D. Weak baby { } E. Prematurity { } F. Nothing { } G. Don’t know { } H. Other (s), specify........
3. What other ways can a pregnant woman prevent herself from getting malaria?

A. Herbal preparations { } B. Sleep under an insecticide treated net { }

C. Mosquito repellent { } E. Use of protective clothing at night { }

F. Taking three doses of Sulphadoxine Pyrimethamine G. Don’t know { } H. Other(s), specify………

**SECTION 4: Perception of respondents about Intermittent Preventive Treatment**

1. How old was your pregnancy when you first attended ANC? A. First month { } B. Second month { } C. Third month D. Forth month { } E. Fifth month { } F. Sixth month { } G. Seventh month { } G. Eighth month { } H. Ninth month { }
2. If 6 months and beyond, why did you attend your first ANC at this time?

A. I could not leave my work { } B. Did not have money for transportation { } C. Did not have any problems during the pregnancy { }

D. Wanted pregnancy to be established culturally { }

E. Long distance to the ANC deterred me { } F. Was being seen by a TBA { } G. Other(s), specify…………

**Section 5: Facility Factors and Practice of DOT**

1. Were you given some medicine by the nurses to swallow before them on your first visit at the ANC? A. Yes { } B. No { }
2. If your answer to question 34 is ‘no’, have you been given any medicines to swallow before the nurses on your subsequent visits? A. Yes { } B. No { }
3. If your answer to question 34 is **‘**yes’, how does the medicine look like? A. Three white tablets in a pack { }. B. Two white tablets in a pack { } C. Loose tablets of different types { } D. One table in a pack
4. How many times during this pregnancy or your last pregnancy did you swallow these same tablets at the Ante-natal clinic (ANC)? A. Once { } B. Twice { } C. Thrice { } D. Can’t remember { }
5. Were you given free, clean water to swallow the medicine? A. Yes { } No { }
6. If your answer to question 38 is ‘no’, how did you get water to swallow the medicine? A. Had my own water { } B. Fetched water from the tap { } C. Bought water at the ANC { } D. Bought water at a pure water seller near the health facility { } E. Other(s), specify ………………………
7. If you were given free water, how was it served? A. In a cup used by other pregnant women { } B. Sachet water (pure water) { } C. In a disposable cup { } D. Used my own cup { }
8. If your answer to question 40 is a cup used by other pregnant women, are you motivated to take the drugs? A. Yes { } B. { }
9. What do you think can be done to motivate you and other pregnant women to return to the health facilities to take subsequent doses of sulphadoxine pyrimethamine?

**Antenatal Record Information**

1. How many times have you visited Ante-natal clinic (ANC) including this one or during your previous pregnancy? { }
2. How old was your pregnancy during your first visit { }
3. Number of doses of Sulphadoxine Pyrimethamine (SP) given (by records) { }
4. Dose of Sulphadoxine Pyrimethamine (SP) given by dates: A.1^st^ B.2^nd^ C.3^rd^ D. None

**Decisions on accessing health facilities**

1. Who is the head of your house hold? A. Father in-law { } B. Mother in-low { } C. Husband D. Brother { } E. Sister { } F. Self { }
2. Who takes decision at home on when to visit the health facility? A. Father in-law { } B. Mother in-low { } C. Husband D. Brother { } E. Sister { } F. Self { }
3. If the answer to ............. is not ‘f’ (self), how do such decisions affect your regular access to health service? A. Not at all { } B. Sometimes { } C. Always { }
4. If it were yourself who takes the decision to visit the health facility, how regular will it be? A. Regular { } B. Irregular { }
5. What motivates you to attend ante-natal clinic? A. Because it is necessary for my health and that of the in-born baby { } B. Family members { } C. Friends

**Staff Attitude**

1. How were you received by the staff on arrival at the health facility? A. Very good { } B. Good { } C. Very poor { } D. Poor { }
2. Did you leave the facility with some questions bothering you that you could not ask? A. Yes { } B. No { }
3. If your answer to question 48 is ‘yes’ why couldn’t you ask? The health worker; A. Shouted at me { } B. Frown the face { } C. was not friendly { } D. Did not have time for me { } E. I did not feel like asking { } E. I was in a hurry { }
4. Did you ask some questions for clarification about the services provided to you in relation to your health and that of the unborn baby A. Yes { } B. No { }
5. If your answer to question 50 is ‘yes,’ what motivated you to ask? The health worker was: A. Friendly { } B. Had enough time for me { } C. Mastered courage to ask because it bothered my mind { }
6. Were your question(s) answered to your satisfaction? A. Yes { } B. No { }
7. Were you ever shouted at during any of your ANC visits? A. Yes { } B. No { }
8. Were you encouraged by the treatment given to attend ANC again? A. Yes { } B. No { }
9. What is your view about the services you received from the health workers A. Satisfactory { } B. Unsatisfactory { }
10. What is your assessment about the behaviour of health workers toward you during your visit at the ante-natal clinic? A. Very good { } B. Good { } C. Bad { } D. Very bad { }
11. Were you encouraged by the treatment you received to tell others to attend Ante-natal clinic? A. Yes { } B. No { }

**Thank you very much for your cooperation**

**Appendix B**

**University of Cape Coast**

**Faculty of Social Sciences**

**QUESTIONNAIRE FOR ANC STAFF**

Questionnaire Code number:

1. Name of facility……………………………………………………
2. Sub-district ………………………………………………………
3. Type of health facility A. Teaching hospital { } B. Regional hospital C. District hospital { } D. Health centre { } E. Ante-natal clinic F. Community based Health Planning Services (CHPS) { }

**SECTION 1: GENERAL INFORMATION**

1. Age: { }
2. Sex: A. Male { } B. Female { }
3. Staff category: A. Midwife { } B. General Nurse { } C. Nurse Assistant { } D. MA { } E. Community Health Nurse E. Other, specify………….......

**If head of unit, please answer questions 6 to 12 in not skip to question 13**

1. Total number of staff required to provide IPT in the facility {}
2. Total number of staff available and providing IPT in the facility {}
3. Number trained on IPT { }
4. Number not trained on IPT { }

Do you have adequate staff to handle clients on IPT and other services

A. Yes B. No

1. Total number of Registrants seen last year
2. Total number of Attendants seen last year
3. Total receiving IPT1.............. IPT2................ IPT3.................... in year 2010

d. Rank of ANC Head …………………………………………………

**SECTION 2: Knowledge about IPTp**

1. What is Intermittent Preventive Treatment of malaria in pregnancy (IPTp)?

........................................................................................................................

1. What is the recommended drug for Intermittent Preventive Treatment of malaria in pregnancy in Ghana?

A. Chloroquine { } B. Artesunate- amodiaquine { } C. Fansidar (SP) { } D. Lumether { } Don’t know { }

1. At what gestation would you not give Sulphadoxine Pyrimethamine during pregnancy? A. Before quickening { } B. After quickening starts { } C. After 36 weeks { } D. between 16 to 35 weeks { } E. Don’t know {} F. Before quickening { }
2. Why would you not give Sulphadoxine Pyrimethamine (SP) at the beginning of a pregnancy?

A. It will cause the woman to vomit { } B. It may have a harmful effect on the foetus { } C. The woman will become weak { } D. It may result to anaemia in the woman and the foetus before quickening starts { } E. Don’t know { }

1. What is the recommended number of doses of Sulphadoxine Pyrimethamine (SP) for IPT during pregnancy in Ghana?

A. Five times { } B. Four times { } C. Thrice { } D. { } Twice { } E. Once { }

F. Don’t know { }

1. At what interval is it recommended that IPT is given? A. Monthly { }

Fortnightly ( ) Every three months ( ) Every week { } Don’t know { }

1. Is Sulphadoxine Pyrimethamine recommended for all pregnant women?

A. Yes { } B. No { }

1. If your answer to question 19 is ‘no’, which category of pregnant women would you not give SP. (tick as many as possible)
2. Does Sulphadoxine Pyrimethamine (SP) have any side effects? A. Yes B. No
3. If your answer to question 21 is ‘yes’, mention some of the side effects
4. Nausea { } B. Skin rash { } B. Vomiting { } C. Other (s) specify...............
5. IPT lessen the incidence of infant and maternal mortality True { } False { }
6. IPT reduces the incidence of low birth weight infants True { } False{ }
7. IPT minimizes the incidence of maternal anaemia True { } False { }
8. Pregnant woman who is allergic to sulphur drugs should be given Sulphadoxine Pyrimethamine True { } False { }
9. Pregnant woman who has received SP less than a month ago is qualified to take Sulphadoxine Pyrimethamine (SP) True { } False { }

**SECTION 3: Practice of directly observed therapy (DOT) for IPTp at ANC**

1. What drug is used for IPTp in your facility? A. Chloroquine { } B. Fansidar { } C. Artesunate-amodiaquine { } D. Lumether { } E. Artesimin Combined therapy { }
2. Do you have Sulphadoxine Pyrimethamine (SP) at the ANC currently? A. Yes { } B. No { }
3. How is Sulphadoxine Pyrimethamine (SP) administered at your facility?

A. The medicine is given to the pregnant women to take home { }

B. Pregnant women are observed to take their medicine at the facility { }

C. Prescriptions are given to pregnant women to collect SP at the pharmacy and take it by themselves { }

D. Prescriptions are given to pregnant women to buy SP outside the facility { }

Other, specify ……………………………………………………

1. Have you ever run short of Sulphadoxine Pyrimethamine (SP) for IPTp in your facility? A. Yes { } B. No { } C. Don’t know { }
2. If your answer to question 31 is ‘yes’, how many times did you experience shortage year 2010? A. Once { } B. Twice { } C. Thrice { } D. more than 3 times { } E. Don’t know { }
3. How did the shortage affect the IPTp programme during that period? A. Suspended till we got the medicine {} B. The women were asked to buy SP { }

C. The women were referred to other Health Facilities { } E. Other(s), specify…………

1. Where is your usual source of supply of SP for IPTp? A. Regional medical stores { }

B. District pharmacist/stores { } C. Health facility itself { } D. Don’t know { } E. Other, specify ……………………………

1. How do pregnant women get clean and safe water to take the SP?

A. Supplied by the facility { } B. Buy from the clinic { } C. Bring from home { } D. Fetch from the tap { } E. Buy from outside the unit { } F. Other(s), specify..........

1. What happens if a pregnant woman attends ANC earlier than when IPTp should be started?

A. All services are given including SP { }

B. Other services are given but excluding Sulphadoxine Pyrimethamine SP { }

C. Other services are given but she is asked come back at quickening or at 16 weeks to start IPTp { }

D. SP is given to her to be taken at home when she is 16 weeks or at quickening ( )

E. Other, specify………………………………………………………

1. Do you provide IPT services at outreach sessions A. Yes { } B. No { }
2. If your answer to question 37 is ‘no’ why...............................................

**SECTION 4: Training in IPTp**

1. Have you ever had any training on IPTp? A. Yes { } B. No { }
2. If yes when was your training or refresher training..............................
3. How many training in IPTp did you receive in the past twelve months?

A. None { } B. Once { } C. Twice { } D. More than twice { } E. can’t remember { }

**SECTION 5: Supervision and Monitoring of IPTP Programme**

1. Did you have any supervisory/monitoring visits at your unit last year?
2. Yes { } B. No { }
3. Was there any monitoring/supervisory visit to your facility last year for IPTp?

A. Yes { } B. No { }

1. If yes, how many times did you have such monitoring/supervisory visits for IPTp? A. Once { } B. Twice { } C. More than twice { } D. Don’t know { }
2. Who did the monitoring/supervision? A. External team { } B. DHMT { } C. Both { } D. Don’t know { }

What do you think can be done to reduce the high dropout rate between intermittent preventive treatment (IPT) 1 and intermittent preventive treatment (IPT) 3? ...........................................................................

**Thank you for your cooperation**

**Appendix C**

**Non participant observation guide**

Name of facility …………………………………………………………

1. Sub-district …………………………………………………………
2. Does health education programme drawn for the quarter include malaria in pregnancy A. Yes { } B. No { }
3. Does health education programme drawn for the quarter include intermittent preventive treatment of malaria in pregnancy (IPTp)

Observations

1. Are there posters of IPTp/MIP posted on the wall?

Observations

1. Is Sulphadoxine Pyrimethamine (SP) available at ANC? (by health workers issuing Sulphadoxine Pyrimethamine to clients).

Observations

1. Are clients swallowing SP before health workers?

Observations

1. Do ANC staff record SP given in ANC book of clients

Observations

1. Is there free safe and clean water for DOT at ANC?

Observations

1. Other observations
